# Enhanced Neutrophil Extracellular Trap Formation in Acute Pancreatitis Contributes to Disease Severity and Is Reduced by Chloroquine

**DOI:** 10.3389/fimmu.2019.00028

**Published:** 2019-01-24

**Authors:** Pranav Murthy, Aatur D. Singhi, Mark A. Ross, Patricia Loughran, Pedram Paragomi, Georgios I. Papachristou, David C. Whitcomb, Amer H. Zureikat, Michael T. Lotze, Herbert J. Zeh III, Brian A. Boone

**Affiliations:** ^1^Division of Surgical Oncology, Department of Surgery, University of Pittsburgh, Pittsburgh, PA, United States; ^2^Department of Pathology, University of Pittsburgh, Pittsburgh, PA, United States; ^3^Center for Biologic Imaging, University of Pittsburgh, Pittsburgh, PA, United States; ^4^Division of Gastroenterology, Hepatology and Nutrition, Department of Medicine, University of Pittsburgh, Pittsburgh, PA, United States; ^5^Department of Surgery, UT Southwestern Medical Center, Dallas, TX, United States; ^6^Department of Surgery, West Virginia University, Morgantown, WV, United States

**Keywords:** neutrophil extracellular traps, pancreatitis, chloroquine, autophagy, systemic inflammatory response, citrullinated histone

## Abstract

**Background:** Neutrophil extracellular traps (NETs) are generated when activated neutrophils, driven by PAD4, release their DNA, histones, HMGB1, and other intracellular granule components. NETs play a role in acute pancreatitis, worsening pancreatic inflammation, and promoting pancreatic duct obstruction. The autophagy inhibitor chloroquine (CQ) inhibits NET formation; therefore, we investigated the impact of CQ mediated NET inhibition in murine models of pancreatitis and human correlative studies.

**Methods:** L-arginine and choline deficient ethionine supplemented (CDE) diet models of acute pancreatitis were studied in wild type and PAD4^−/−^ mice, incapable of forming NETs. Isolated neutrophils were stimulated to induce NET formation and visualized with fluorescence microscopy. CQ treatment (0.5 mg/ml PO) was initiated after induction of pancreatitis. Biomarkers of NET formation, including cell-free DNA, citrullinated histone H3 (CitH3), and MPO-DNA conjugates were measured in murine serum and correlative human patient serum samples.

**Results:** We first confirmed the role of NETs in the pathophysiology of acute pancreatitis by demonstrating that PAD4^−/−^ mice had decreased pancreatitis severity and improved survival compared to wild-type controls. Furthermore, patients with severe acute pancreatitis had elevated levels of cell-free DNA and MPO-DNA conjugates, consistent with NET formation. Neutrophils from mice with pancreatitis were more prone to NET formation and CQ decreased this propensity to form NETs. CQ significantly reduced serum cell-free DNA and citrullinated histone H3 in murine models of pancreatitis, increasing survival in both models.

**Conclusions:** Inhibition of NETs with CQ decreases the severity of acute pancreatitis and improves survival. Translating these findings into clinical trials of acute pancreatitis is warranted.

## Introduction

Acute pancreatitis (AP) is a common, severe gastrointestinal disease, assessed as the fifth leading cause of in-hospital mortality and an annual incidence that has increased by 30% since 2000 (1, 2). AP is characterized by elevated digestive enzyme concentrations in the blood linked to altered acinar cell secretion and/or duct obstruction, which promotes autodigestive injury within the pancreas (2). These events stimulate tissue injury and activation of the innate immune system, resulting in recruitment and activation of neutrophils with subsequent release of pro-inflammatory cytokines and other substances that lead to systemic inflammation (2, 3). The pathogenesis of severe AP is still poorly understood, limiting significant treatment options outside of supportive care including bowel rest, fluid resuscitation, and pain management (1, 2).

Neutrophils play a central role in severe acute pancreatitis. In addition to release of cytokines, activated neutrophils release their DNA, histone proteins, high mobility group box 1 (HMGB1), and granule components into the extracellular space or circulation to form neutrophil extracellular traps (NETs). This process was initially considered primarily as a means for neutrophils to trap bacteria and combat infection (4–6). Although NETs are generally immuno-protective in the setting of bacterial or fungal infections (4), several studies have implicated NETs in the pathophysiology of sterile inflammatory conditions, such as lupus (7, 8), rheumatoid arthritis (9), and pancreatic cancer (10). Recent evidence demonstrates that NETs are also involved in the pathogenesis of AP by inducing trypsin activation, inflammation, and tissue damage (11) following inspissation and occlusion of pancreatic ducts (12). Moreover, NETs surround necrotic tissue in patients with severe AP and promote the subsequent systemic inflammatory response syndrome (13).

Chloroquine (CQ), is an orally available and inexpensive drug historically used to treat malaria (14) that also inhibits autophagy (15). We and others have shown that heightened autophagy promotes NET generation (16–19) and that CQ prevents NET formation (10, 20). Given the recent discovery of the role of NETs in AP, we studied whether targeted inhibition of NETs with CQ could ameliorate AP in murine models. We evaluated the role of CQ in the treatment of acute pancreatitis by observing its effect on NET formation, and impact on the severity and survival rate in murine models of pancreatitis. Furthermore, we performed correlative studies on the impact of NETs on the severity of human pancreatitis.

## Materials and Methods

### Murine Models and Treatments

All experimental procedures were approved by the Institutional Animal Care and Use Committee of the University of Pittsburgh (Protocol # 14084123) and performed in accordance with the policies and regulations established by the University of Pittsburgh Division of Laboratory Animal Services, the Guide for the Care and Use of Laboratory Animals, and the Animal Research Reporting on *in vivo* Experiments (ARRIVE) guidelines. Euthanasia was performed under anesthesia using cardiac puncture resulting in exsanguination followed by cervical dislocation. Mice were housed in ventilated caging units in the Hillman Cancer Center Specific Pathogen Free (SPF) animal facility with standard housing, husbandry, and free access to food and water. C57/BL6 wild type mice (4 and 10–12 weeks) were purchased from Taconic Farms (Hudson, New York). PAD4 knockout (PAD4^−/−^) mice, incapable of forming NETs, were obtained as a kind gift from the late Dr. Kerri Mowen (21) and were generated on a C57/Bl6 background. Induction of AP using L-arginine (22) or choline deficient ethionine (CDE) supplemented diet (23) was performed as previously described in age and gender matched mice (24). Briefly, a sterile solution of 8% L-arginine hydrochloride (A92600, Millipore Sigma, Burlington, MA) was prepared in normal saline and adjusted to pH 7.0. Mice received 2 hourly intraperitoneal (IP) injections of L-arginine (4 g/kg), while controls were administered saline IP. Animals were treated with oral chloroquine (CQ) (0.5 mg/ml) administered in the drinking water *ad libitum* upon completion of second L-arginine injection. Isoflurane anesthetized mice were sacrificed via cardiac puncture at 48 or 72 h post injection. Serum was collected after blood was allowed to clot for 30 min and then spun at 10,000 g for 10 min.

For survival experiments, age and gender matched mice underwent two intra-peritoneal L-arginine (4 g/kg) injections an hour apart once a week for a total of 3 weeks. Survival was assessed over a 6 week period. A choline deficient ethionine (CDE) supplemented diet model of AP was also utilized as previously described (23, 25). Briefly, 4 week-old female mice were fasted for 24 h and then fed a CDE diet (960214, MP Biomedicals, Solon, OH) for 6 days. For CDE experiments, animals were treated with oral CQ (0.5 mg/ml) administered in the drinking water *ad libitum* at the start of the CDE diet (CDE CQ).

### Human Samples

Blood was collected from patients with acute pancreatitis as part of a protocol approved by the Institutional Review Board at the University of Pittsburgh (#PRO08010374, PRO14060166). Severity of acute pancreatitis was classified by the revised Atlanta classification (26). Blood samples were drawn within 72 h of presentation, spun at 14,000 g for 10 min and serum was collected and frozen at −80°C using strict standard operating protocols as previously described (27). Serum samples from 5 healthy volunteers were also evaluated as controls.

### Biochemical Pancreatitis and Systemic Inflammatory Assays

Trypsin and amylase activity levels, HMGB1, and interleukin-6 (IL-6) levels in murine serum diluted 1:10 were measured using ELISA and quantified using a Tecan Saphire microplate reader. The colorimetric mouse trypsin activity ELISA assay (E4362-100, BioVision, San Francisco, CA), mouse amylase assay kit (ab102523, Abcam, Cambridge, MA), human/mouse HMGB1 ELISA (ST51011, IBL International, Hamburg, Germany), and mouse IL-6 uncoated ELISA (88-7064, Invitrogen, Carlsbad, CA) were used according to manufacturer protocols.

### *Ex vivo* NET Formation and Quantification

Under sterile conditions, bone marrow neutrophils were isolated from the femur and tibia of euthanized mice by the previously described protocol (28). Neutrophils were plated in a 24-well plate at 1.5 × 10^4^ cells per well in Hank's Balanced Salt Solution (14025076, ThermoFisher Scientific, Waltham, MA). Neutrophils were then stimulated with 40 μm, platelet activating factor (PAF) (511075, Millipore Sigma) for 120 min. Cells were fixed with 3% paraformaldehyde and stained for DNA with Hoechst 33342 (H-3570, Molecular Probes, Grand Island, NY). Representative neutrophils were stained for citrullinated histone H3 (anti-Histone H3 (citrulline 2 + 8 + 17) antibody, Abcam, Cambridge, MA) to confirm that NETs were being visualized rather than DNA released from necrosis. NETs were visualized using a Zeiss Axiovert 40 microscope under 10x−40x magnification. Supernatant was collected, spun at 14 g for 10 min and the level of DNA measured using Quant-iT Picogreen (MP07581, Invitrogen, Grand Island, NY).

### Measures of *in vivo* NET Formation

After 1:10 dilution, murine serum samples were measured for cell-free DNA (cf-DNA) with Quant-iT Picogreen (MP07581, Invitrogen) and citrullinated histone H3 was measured with the citrullinated histone H3 ELISA (501620, Cayman Chemical, Ann Arbor, MI) according to the manufacturer's protocol. Cell-free DNA was measured in the patient serum with the Quant-iT Picogreen (MP07581, Invitrogen). To show that circulating nucleosomes in sera are derived from NETs, we tested myeloperoxidase, a prominent granular component of neutrophils, attached to nucleosomes as described previously (29). MPO-DNA complexes were identified using a capture ELISA (component No.1, Hycult biotech, HK210-01, Uden, Netherlands). A 1:2 dilution of sample was added to the wells and incubated for 1 h. After washing three times, 100 μl incubation buffer containing a peroxidase-labeled anti-DNA mAb (component No.2, Cell Death ELISA^PLUS^, Roche; Cat. No: 11774424001, Basel, Switzerland) was used according to the manufacturer's protocol.

Resected pancreatic tissue was fixed in OCT medium and stored in −80°C until further analysis. After sectioning to 6 μm, the pancreas section was permeabilized with 0.1% Triton X-100 in Phosphate Buffered Saline (PBS) for 15 min, blocked with 20% normal donkey serum (NDS) for 45 min, and washed 1x with 0.5% BSA in PBS. The sections were incubated with anti-Histone H3 (Rabbit, 1:200, citrulline R2 + R8 + R17, Abcam, ab5103) and anti-Ly6G (Rat, 1:100, RB6-8C5, Invitrogen). After three washes, donkey anti-rabbit Alexa 488 (A21206, 1:500, Jackson ImmunoResearch Laboratories, West Grove, PA) to recognize the anti-Histone H3 was combined with the donkey anti-rat Cy3 IgG-Cy3 conjugated (712-165-153, 1:1000, JacksonImmunoResearch Laboratories, West Grove, PA) that paired with the Ly6G were both incubated for 1 h. A Hoechst nuclear stain (B-2883, Millipore Sigma) was applied at room temperature for 30 s followed by a single rinse of PBS to remove excess dye. Imaging conditions were maintained at identical settings within each antibody-labeling experiment with original gating conditions compared to sample that was a “primary delete,” processed with all reagents except primary antibody. Imaging was performed using a Nikon A1 confocal microscope (purchased with 1S10OD019973-01, awarded to Dr. Simon C. Watkins) at 20x and analyzed with Nikon Elements imaging software (NIS Elements 4.4, Tokyo, Japan).

### Statistical Analysis

Data are expressed as mean ± standard deviation. Results are reported from at least two independent experiments performed with at least triplicate samples. Statistical analysis was performed using Student's two tailed *t-*test for comparisons of two groups or 1-way ANOVA with Tukey's *post-hoc* tests for multiple groups (GraphPad Prism, San Diego, CA, United States). Survival analyses were conducted using the Kaplan-Meier method and Grehan-Breslow-Wilcoxon test between curves. *p* < 0.05 were considered statistically significant.

## Results

### NETs Are a Critical Mediator of Murine and Human Acute Pancreatitis

**Figure 1:**
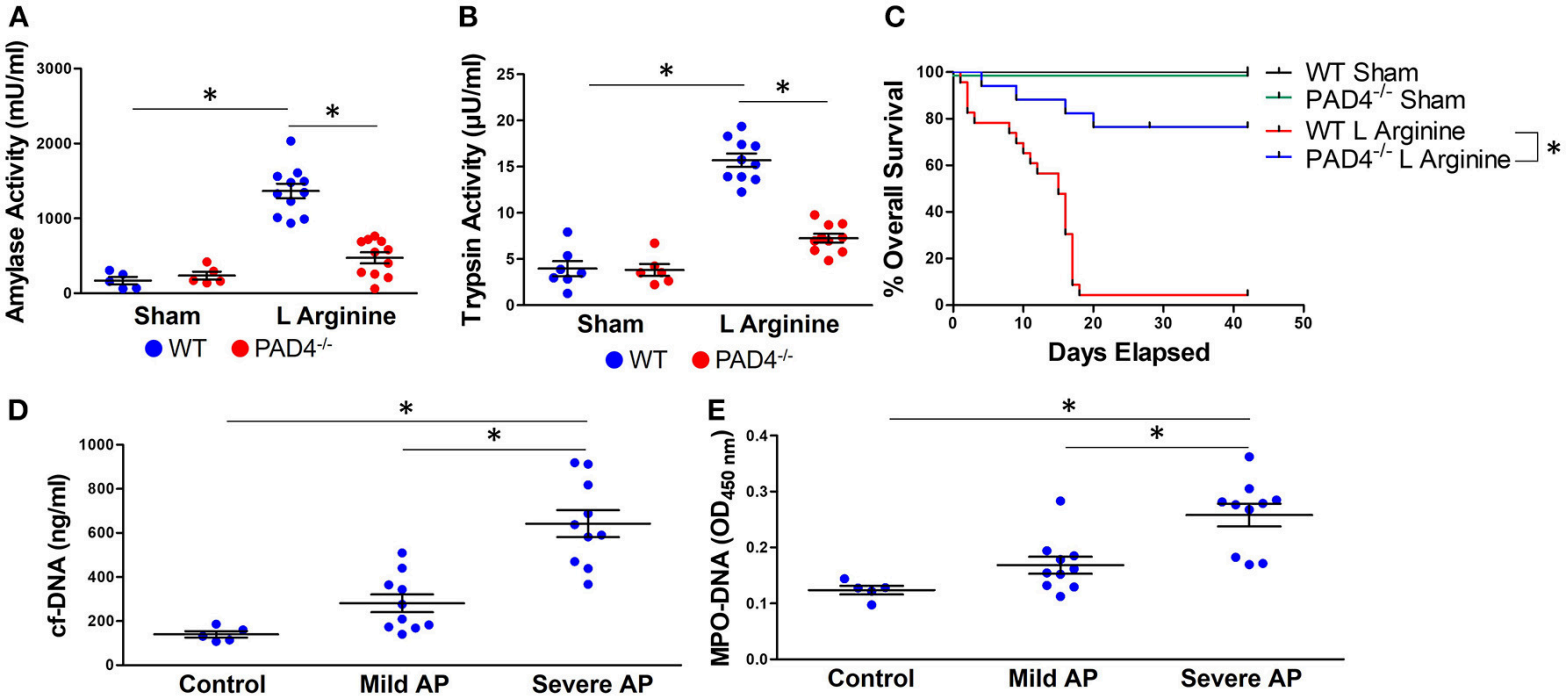
NETs are critical mediators of murine and human acute pancreatitis (AP). Induction of pancreatitis with L-arginine resulted in an increase in amylase **(A)** and trypsin **(B)** activity in wild-type mice that was statistically diminished in PAD4^−/−^ mice, incapable of forming NETs. PAD4^−/−^ mice had significantly improved survival compared with wild type mice in recurrent L-arginine induced murine acute pancreatitis (median survival unreached vs. 15 days, *p* < 0.0001, *n* = 12 per group) **(C)**. Patients with pancreatitis had elevated levels of circulating cell-free DNA **(D)** and MPO-DNA conjugates **(E)**, biomarkers of NET formation that correlated with disease severity based on the revised Atlanta classification. ^*^p < 0.05.

To confirm the impact of NET formation on the pathophysiology of severe AP, we utilized mice unable to form NETs due to a genetic ablation of protein arginine deiminase, type IV (PAD4), an enzyme responsible for chromatin decondensation and subsequent expulsion during NETosis (30). PAD4^−/−^ mice with pancreatitis had decreased trypsin (7.25 ± 1.5 vs. 15.7 ± 2.3 μU/ml, *p* < 0.0001) and amylase (472 ± 242 vs. 1365 ± 322 μU/ml, *p* < 0.0001) activity levels compared to wild-type controls (Figures 1A,B). To evaluate the impact of NETs on survival from severe acute pancreatitis, mice were injected with L-arginine weekly for 3 weeks. Survival was significantly improved in PAD4^−/−^ mice with pancreatitis compared to wild-type controls (median survival unreached vs. 15 days, *p* < 0.0001, Figure 1C).

We next investigated the clinical relevance of NETs in patients with AP. Serum was isolated from patients with mild or severe acute pancreatitis, as determined by the revised Atlanta classification (26). Patient demographics and clinical characteristics are reported in Supplemental Table 1. Serum cell-free DNA (642 ± 193 vs. 281 ± 127 and 140 ± 32 ng/ml, *p* < 0.001, Figure 1D) and MPO-DNA complexes, markers of NET formation, were significantly elevated in patients with severe AP in comparison with mild AP and healthy controls (0.258 ± 0.06 vs. 0.168 ± 0.05 and 0.123 ± 0.01 OD_450_, *p* < 0.001, Figure 1E). There was a significant correlation between levels of cf-DNA and MPO-DNA (Supplemental Figure 1, Pearson *r* = 0.868, 95% CI: 0.719–0.940, *p* < 0.0001).

### Chloroquine Inhibits NET Formation in Acute Pancreatitis

**Figure 2:**
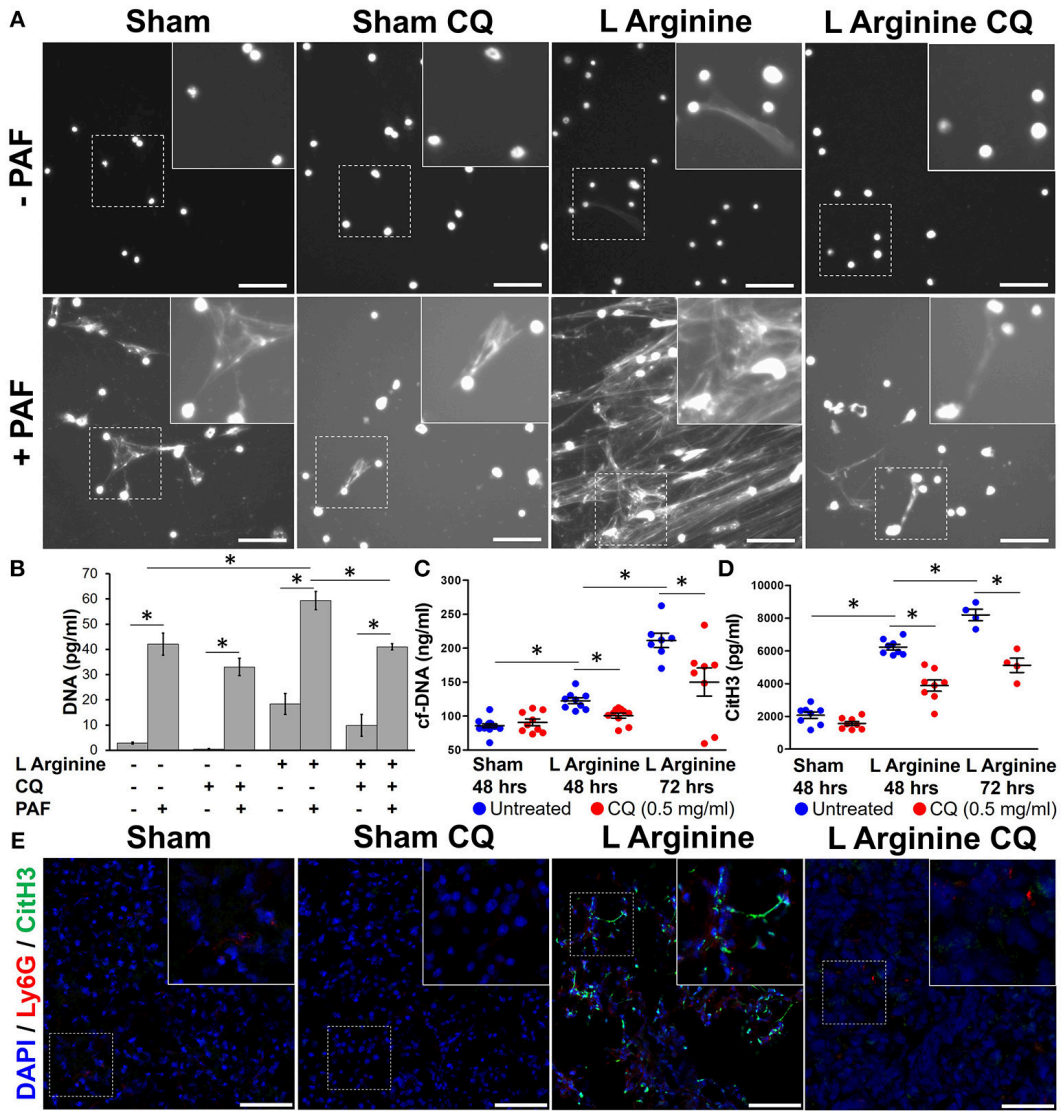
Chloroquine inhibits NET formation in acute pancreatitis. Neutrophils harvested from mice with pancreatitis had evidence of spontaneous NET formation and greater propensity to form NETs upon stimulation with platelet activating factor (PAF), as visualized by staining of DNA with Hoechst **(A)**. CQ treatment dramatically reduced spontaneous and stimulated NET formation. Supernatant DNA was measured to objectively quantify NETs **(B)**. Serum cell-free DNA **(C)** and citrullinated histone H3 (CitH3) **(D)**, biomarkers of *in vivo* NET formation were elevated with induction of pancreatitis, but significantly reduced with chloroquine treatment. Co-localization of pancreatic CitH3 (Green), with neutrophils (Ly-6G, Red) is increased in pancreatitis mice compared to sham controls **(E)**, but treatment with CQ reduced neutrophil CitH3 expression (20x, scale bar = 50 μm). These are representative images from at least two independent analyses. ^*^p < 0.05.

Isolated bone marrow neutrophils from wild type mice were plated and stained with Hoechst to evaluate their propensity to form NETs. As previously described (10), citrullinated histone H3 was identified within NETs on this *ex vivo* assay (Supplemental Figure 2), confirming that DNA visualized from neutrophils was due to NET formation and not cell necrosis. At baseline, untreated neutrophils from sham control mice produced no NETs. Neutrophils from mice with L-arginine induced pancreatitis produced spontaneous NETs (Figure 2A). Isolated neutrophils were stimulated with PAF, a known inducer of NET formation. Mice with pancreatitis had a greater propensity for NET formation, demonstrated by numerous large, intricately connected NETs. Treatment with CQ markedly decreased the propensity to form NETs in response to PAF stimulation and prevented spontaneous NET formation in mice with pancreatitis.

To objectively quantify the increased extracellular DNA visualized during *ex vivo* NET formation, supernatant cell-free DNA was measured. L-arginine pancreatitis resulted in a 6-fold increase in cell-free DNA compared to sham controls (18.4 ± 4.2 vs. 2.9 ± 0.4 ng/ml, *p* = 0.0004), indicative of increased NET formation during AP (Figure 2B). Treatment of CQ in pancreatitis mice decreased supernatant cell-free DNA levels in unstimulated (9.87 ± 4.3 vs. 18.37 ± 4.2 ng/ml, *p* = 0.03) and PAF stimulated neutrophils (40.99 ± 1.2 vs. 59.39 ± 3.6 ng/ml, *p* < 0.0001).

*In vivo* NET formation was next assessed in mice with severe acute pancreatitis. Serum levels of circulating cf-DNA were measured as a biomarker of NET formation (10). Serum cf-DNA was elevated in pancreatitis mice and was decreased with CQ treatment after 48 h (100 ± 11 vs. 122 ± 12 ng/ml, *p* = 0.0011, Figure 2C) and after 72 h (211 ± 28 vs. 115 ± 59 ng/mL, *p* < 0.001). Citrullinated histone 3 (CitH3), one of the histone proteins formed and released during NET formation, was elevated in pancreatitis at 48 h (6,228 ± 471 vs. 2,071 ± 564 pg/ml, *p* < 0.0001, Figure 2D) and further increased at 72 h (8,198 ± 700 vs. 6,228 ± 471 pg/ml, *p* = 0.0002). CitH3 levels correlated significantly with cf-DNA (Pearson *r* = 0.676, 95% CI 0.375–0.848, *p* = 0.0003). Treatment with CQ significantly reduced CitH3 levels at both time points (3,878 ± 962 vs. 6,228 ± 471 pg/ml, *p* < 0.0001 and 5,120 ± 876 vs. 8,198 ± 700 pg/ml, *p* = 0.0015). To assess for NET formation in the pancreas, CitH3 expression was evaluated in resected murine pancreatitis sections after L-arginine injection. Pancreatic neutrophil infiltration, as assessed by neutrophil Ly6g, and CitH3 was increased in pancreatitis mice compared to sham controls (Figure 2E). Unmerged images are available for review in Supplemental Figure 3. CitH3 expression co-localized with Ly6g, a neutrophil GPI-anchoring protein, suggesting that CitH3 was released from infiltrating neutrophils. Treatment of CQ markedly reduced pancreatic neutrophil infiltration and NET formation, as demonstrated by decreased CitH3/Ly-6g co-localization (Figure 2E).

### Chloroquine Treatment Reduces Local and Systemic Inflammation From Pancreatitis

**Figure 3:**
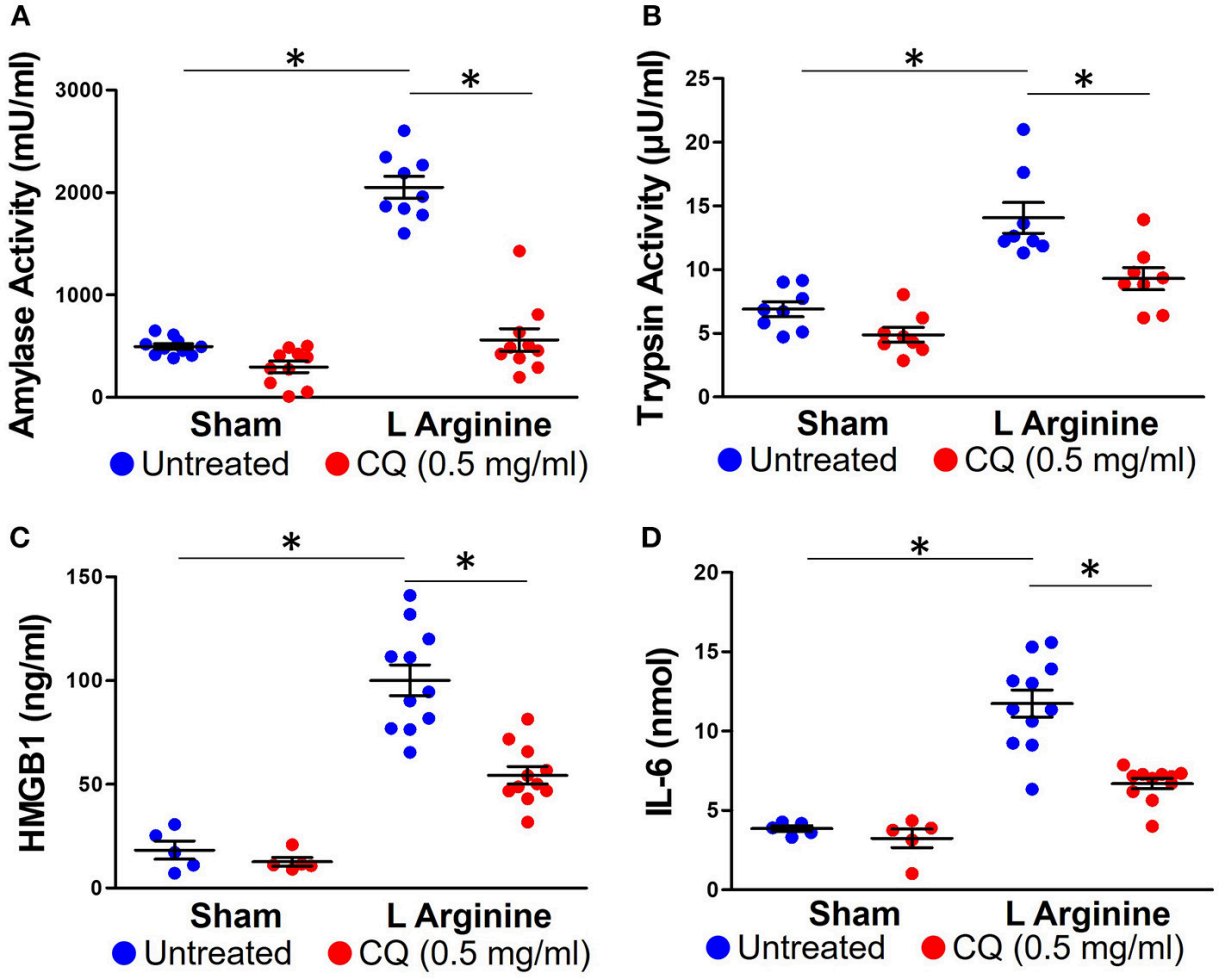
Chloroquine reduces local and systemic inflammation in acute pancreatitis. Chloroquine treatment led to a significant reduction in amylase **(A)** and trypsin **(B)** activity. The systemic inflammatory response to acute pancreatitis was assessed by measuring HMGB1 **(C)** and IL-6 **(D)**. Chloroquine treatment led to a significant reduction in both of these circulating inflammatory markers. ^*^p < 0.05.

Treatment with CQ in mice with pancreatitis reduced amylase (561 ± 348 vs. 2,051 ± 320 mU/ml, *p* < 0.0001) and trypsin activity (5.58 ± 1.5 vs. 8.44 ± 2.1 μU / ml, *p* = 0.006) (Figures 3A,B). As part of the systemic inflammatory response to AP, activated neutrophils and other leukocytes release pro-inflammatory compounds, further propagating local and systemic inflammation. Serum HMGB1 and IL-6 significantly increased with L-arginine induction of pancreatitis (95.9 ± 21.7 vs. 18.2 ± 9.7 ng/ml, *p* < 0.0001; 11.66 ± 2.82 vs. 3.85 ± 0.04 nmol) (Figures 3C,D). Administration of CQ significantly reduced serum HMGB1 (54.35 ± 14.7 vs. 95.99 ± 21.7 ng/ml, *p* < 0.0001, Figure 3C) and IL-6 (6.69 ± 1.07 vs. 11.66 ± 2.82 nmol, *p* < 0.0001, Figure 3D).

### Chloroquine Treatment Improves Survival in Severe Acute Pancreatitis

**Figure 4:**
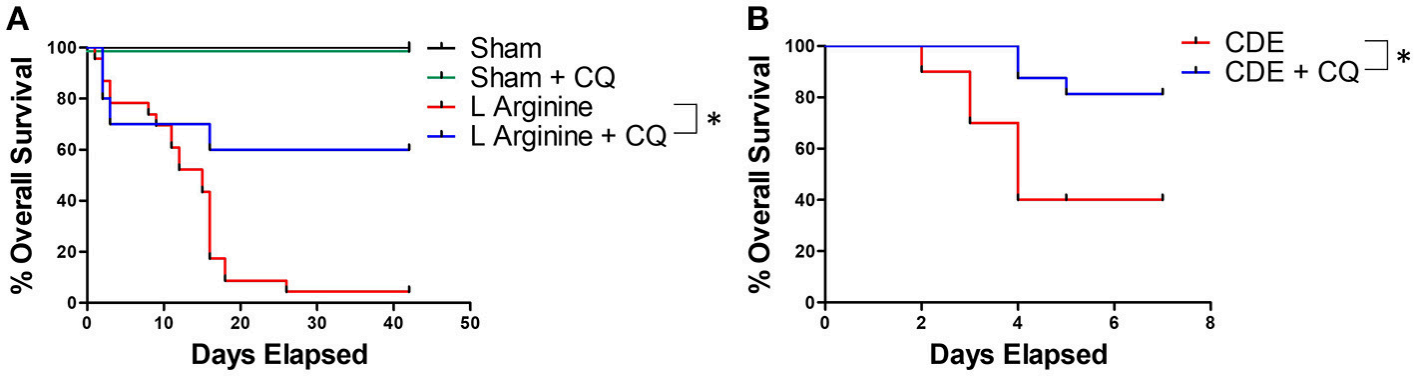
NET inhibition with chloroquine improves survival in murine acute pancreatitis. Chloroquine treatment led to significantly improved survival in murine severe acute pancreatitis (**A**, median survival unreached vs. 15 days, *p* = 0.0001, *n* = 10 per group). These profound findings were confirmed in a second model of murine pancreatitis utilizing administration of choline deficient, ethionine (CDE) supplemented diet (**B**, median survival unreached vs. 4 days, *p* = 0.016, *n* = 20 per group). ^*^p < 0.05.

In a survival model of recurrent L-arginine induced pancreatitis, CQ treatment significantly improved survival compared to untreated mice (median survival unreached vs. 15 days, *p* = 0.0001, Figure 4A). To confirm these findings in another model of murine acute pancreatitis, a choline deficient, ethionine (CDE) supplemented diet was also utilized to test the efficacy of CQ treatment. Treatment with CQ also significantly improved survival in CDE diet induced pancreatitis (median survival unreached vs. 4 days, *p* = 0.016, Figure 4B). Importantly, CQ did not have a significant effect on survival from severe acute pancreatitis in PAD4^−/−^ mice, suggesting that NET inhibition may be a central mechanism for CQ mediated improvement in survival (Supplemental Figure 4).

## Discussion

Acute pancreatitis (AP) is the most common disease involving the gastrointestinal tract with a mortality rate in the most severe cases, ranging from 20 to 30% (31). This mortality rate has not improved in the last three decades. No effective therapies have been developed for patients with AP outside of supportive care, highlighting the critical need for novel therapies that target the underlying pathophysiology of the disease (1). Neutrophils are critical to the development of severe AP, further promoting trypsinogen activation, recruitment of immune cells to the pancreas, and propagating tissue inflammation and damage (3). In the studies reported here, we demonstrate that NETs correlate with the severity of murine and human acute pancreatitis and that treatment with the drug chloroquine inhibits NETs and is associated with improved outcomes for severe AP.

Neutrophil extracellular traps (NETs) have emerged as a critical mediator of the innate immune response to sterile inflammatory disease (4, 7–9, 16). NETs have been identified in the pathogenesis of AP (11–13). In murine pancreatitis induced following taurocholate injection, NETs were identified within the pancreatic tissue using scanning electron microscopy to visualize co-localized DNA, histones and neutrophil elastase (11). NETs were also shown to induce trypsin activation (11). Aggregates of NET components occlude pancreatic ducts in murine IL-17 induced pancreatitis to propagate pancreatic inflammation (12). Importantly, protein arginine deiminase, type IV (PAD4), an enzyme required for NET formation that citrullinates histones to allow for decondensation, was necessary for induction of pancreatitis in this model. Herein, we expand on previous findings that NETs are a critical mediator of acute pancreatitis in two additional models of murine pancreatitis; using L-arginine (22) and a choline deficient, ethionine supplemented (CDE) diet (23) to induce pancreatitis. We found that pancreatitis primes neutrophils to increase their propensity to form NETs in an *ex vivo* assay. Furthermore, PAD4^−/−^ mice, incapable of NET formation, had reduced severity and improved survival compared to wild-type controls, implicating NETs in the pathophysiology of severe pancreatitis in these models.

Because no murine model perfectly recapitulates the multiple etiologies driving human pancreatitis, establishing the relevance of NETs in human disease is critical. NET aggregates have been visualized in pancreatic ducts from human benign and malignancy associated pancreatitis (12). Patients with pancreatitis have elevated levels of NET biomarkers including cell-free DNA and DNA-histone conjugates compared with controls (11). We expanded these observations to demonstrate that NET biomarkers are not only present in human pancreatitis, but also correlate with severity of disease. While cell free DNA is non-specific for NETs and may be released from necrotic cells in necrotizing pancreatitis, MPO-DNA conjugates are a much more specific bio-marker for NET formation (32). We have also shown evidence of NETs in the pancreatic tissue during acute pancreatitis, with the immunofluorescence staining of citrullinated histone H3. While a portion of this expression does not co-localize with the neutrophil marker Ly6G, this could be due to citrullinated histone deposition from NETs in the extracellular space with clearance of Ly6G after the neutrophil dies during NETosis. Additionally, citrullination of histone H3 is a fairly specific marker of NET formation, therefore these findings do suggest NETs occur within the pancreatic tissue during acute pancreatitis. Taken together, these data suggest that NETs are a clinically relevant therapeutic target, since systemic induction of NETs are likely to contribute to the multi-system organ dysfunction that defines severe acute pancreatitis (6, 26, 33, 34).

Based on the evidence that NETs appear to be critical to the pathogenesis of murine severe acute pancreatitis and strongly associated with human severe acute pancreatitis, we evaluated NET inhibition as a novel therapeutic target to improve outcomes for patients with this disease. Strategies to deplete neutrophils or block adhesion molecules to limit neutrophil infiltration have limited tissue damage in acute pancreatitis (35–39). However, our understanding of the molecular mechanisms responsible for NET formation, allows for development of therapies to specifically target this process. Administration of DNase for NET inhibition prior to induction of acute pancreatitis resolved pancreatic tissue damage and decreased systemic inflammation in taurocholate and L-arginine induced murine acute pancreatitis (11). DNase lyses extracellular DNA released from neutrophils to prevent accumulation of NET aggregates. As a “late” inhibitor of NET formation, DNase treatment does not prevent NET formation and does little to counter-act the effects of the release of other NET components, including histones, HMGB1, elastase and other factors (40). PAD4 is an enzyme that is essential for NETosis. PAD4 inhibitors have been developed that prevent NETs and warrant further exploration in pancreatitis (41–44). Interestingly, both the receptor for advanced glycation end productions (RAGE) and HMGB1 have been studied in the development of pancreatitis (45–48) and linked to the promotion of NET formation(10, 49). This association suggests that they may exert their inflammatory effects in pancreatitis through induction of NETosis and may represent additional upstream targets for NET inhibition.

Chloroquine (CQ) is an inexpensive drug with an established safety and toxicity profile that has been discovered to inhibit NETs in pancreatic cancer, most likely through the inhibition of neutrophil autophagy, which is required for NET formation (10, 50). Chloroquine has been utilized for many years to treat patients with malaria, systemic lupus erythematosus, and rheumatoid arthritis, but has also been evaluated previously as a treatment for acute pancreatitis (51–55). Leach et al. investigated the treatment of CQ in a choline-deficient ethionine-supplemented (CDE) diet model of severe acute murine pancreatitis. CQ pre-treatment neutralized the subcellular pH of lysosomes and decreased 72 h serum amylase and trypsin activity associated with the diet-induced pancreatitis (54). These findings were supported by the work of Guillaumes et al. who showed that high-dose CQ administration improved 1-week survival in murine CDE diet induced pancreatitis from 40 to 70% (53). Despite these studies supporting the benefit of CQ in acute pancreatitis, there have also been contradictory findings (55). Our findings validate the efficacy of CQ as a treatment for AP in CDE diet induced pancreatitis and another murine model, L-arginine induced pancreatitis. CQ reduced the serum biochemical activity and levels of amylase and trypsin and also reduced systemic levels of inflammatory cytokines HMGB1 and IL-6. Importantly, CQ improved survival in two different murine models of severe acute pancreatitis.

Previously, CQ efficacy in AP was thought to be mediated by stabilization of lysosome function or by inhibiting acidification of the lysosomes to prevent activation of trypsinogen and digestive zymogens (52–54). However, controversy regarding the precise mechanism of CQ in pancreatitis remains (56). We identified inhibition of NET formation as another potential mechanism for the beneficial effects of CQ in pancreatitis. In the *ex vivo* NET assay, CQ treatment notably prevented spontaneous NET formation, and reduced the propensity to form NETs upon neutrophil stimulation. CQ treatment also lowered *in vivo* NET formation, reducing serum cell-free DNA and CitH3, a NET biomarker that allows for unwinding and expulsion of neutrophil DNA during NET formation. Immunolabeling of murine pancreatitis specimens showed that CQ treatment also decreased infiltration of neutrophils in the pancreas and pancreatic neutrophil-CitH3 co-localization. Importantly, CQ inhibits autophagy, a cell survival mechanism that utilizes degradation and recycling of damaged intracellular materials and is required for NET formation (17, 57–60), possibly by alkalinization of lysosomes (54). More recently, autophagy itself has been implicated in the pathogenesis of AP (61, 62). Other studies suggest that it is impaired, blocked, or disordered autophagy within acinar cells that initiates pancreatitis in multiple models of AP, and that restoration of autophagic flux and clearance of protein aggregates abrogates pancreatitis in these models (63). CQ also inhibits the function of activated platelets (64, 65), which are a critical mediator of the inflammatory response during acute pancreatitis (66–69). Taken together, these finding suggests that CQ, in adequate doses, minimizes the activation of trypsinogen to trypsin in the pancreatic acinar cell during cell stress (initiation of AP), and also limits progression to severe acute pancreatitis through effects on neutrophils and/or other inflammatory cells. While we demonstrate that CQ reduces NETs and that CQ treatment is associated with improved inflammatory outcomes and survival in murine acute pancreatitis, there are multiple other potential mechanisms through which CQ may be functioning, including those outlined above. In an attempt to demonstrate NET inhibition as the primary mechanism for CQ in pancreatitis, we treated PAD4^−/−^ mice with CQ and did not observe any difference in survival as compared to wild type mice treated with CQ. This strengthens the association between CQ induced improvement in survival and NET inhibition as there is less impact for CQ when NETs are genetically diminished, however a direct causal mechanism is difficult to demonstrate. Therefore, we must interpret our results on the mechanism of CQ in acute pancreatitis and association with NET inhibition with caution.

This work is limited by the fact that no murine model of experimental pancreatitis perfectly recapitulates human disease. In consideration of this, we utilized two different murine models to demonstrate the survival benefit associated with CQ treatment. Additionally, we evaluated patient correlates to demonstrate that NETs are associated with human severe acute pancreatitis. In the current study, we initiated CQ shortly after initiation of pancreatitis to explore it as a rescue strategy for ill patients, however given the evidence that NETs may contribute to the early development of acute pancreatitis, NET inhibition should also be explored as a possible prophylactic therapeutic treatment strategy for patients at high risk for AP.

In conclusion, NETs have been identified as a key component in the pathophysiology of murine and human acute pancreatitis, driving severity and mortality, and serve as a therapeutic target to improve outcomes in AP. Chloroquine inhibits NET formation and ameliorates murine acute pancreatitis. Further study into the targeted inhibition of NETs with CQ in patients with severe acute pancreatitis or in patients at risk for recurrent pancreatitis is warranted, as well as studies designed to develop parenterally administered congeners.

## Data Availability Statement

The raw data supporting the conclusions of this manuscript will be made available by the authors, without undue reservation, to any qualified researcher.

## Author Contributions

PM, ML, HZ, BB, GP, DW, and PP: study concept and design. PM, AS, MR, PL, PP, DW, GP, and BB: acquisition, analysis, and interpretation of data. PM, BB, PL, and MR: drafting of the manuscript. PM, AS, MR, PL, PP, GP, DW, AZ, ML, HZ, and BB: critical revision of the manuscript. AS, GP, DW, AZ, ML, HZ, and BB: funding, administrative, or material support.

### Conflict of Interest Statement

The authors declare that the research was conducted in the absence of any commercial or financial relationships that could be construed as a potential conflict of interest.

## Abbreviations

AP: acute pancreatitis
CQ: chloroquine
NETs: neutrophil extracellular traps
CitH3: citrullinated histone H3
cf-DNA: cell free-DNA
IL-6: interleukin-6
PAF: platelet activating factor.
