# Supplementary material for: Enhanced Neutrophil Extracellular Trap Formation in Acute Pancreatitis Contributes to Disease Severity and Is Reduced by Chloroquine
Source: Front Immunol. 2019 Jan 24;10:28. doi: 10.3389/fimmu.2019.00028 (PMC6353831; doi:10.3389/fimmu.2019.00028)
Supplement: Supplementary file 1 [file Table_1.docx]

| **Patient Demographics** | | | |
| --- | --- | --- | --- |
| **Age** | | 58 (25-95) | |
| **Gender** | |  | |
|  | Male | 9 (45%) | |
|  | Female | 11 (55%) | |
| **BMI** | | 30 (21-45) | |
| **CCI** | | 1 (0-5) | |
| **Etiology** | |  | |
|  | Gallstone | 16 (80%) | |
|  | Alcohol | 0 (0%) | |
|  | Idiopathic | 1 (5%) | |
|  | Hypertriglyceridemia | 1 (5%) | |
|  | Post-ERCP | 1 (5%) | |
|  | Other | 1 (5%) | |
| **Episode of pancreatitis** | | | |
|  | First bout | | 16 (80%) |
|  | Recurrent | | 4 (20%) |
| **Length of Stay (days)** | | | 7 (3-80) |
|  |  |  | |
| **Supplemental Table 1**: Demographics of patients with acute pancreatitis studied. Data reported as median (range) or n(%). BMI= Body mass index. CCI= Charlson co-morbidity index.  The Supplementary Material for this article can be found online at: https://www.frontiersin.org/articles/10.3389/fimmu.  2019.00028/full#supplementary-material | | | |
|  | |  | |
|  | | | |
