# Supplementary figures and images for: Enhanced Neutrophil Extracellular Trap Formation in Acute Pancreatitis Contributes to Disease Severity and Is Reduced by Chloroquine
Source: Front Immunol. 2019 Jan 24;10:28. doi: 10.3389/fimmu.2019.00028 (PMC6353831; doi:10.3389/fimmu.2019.00028)

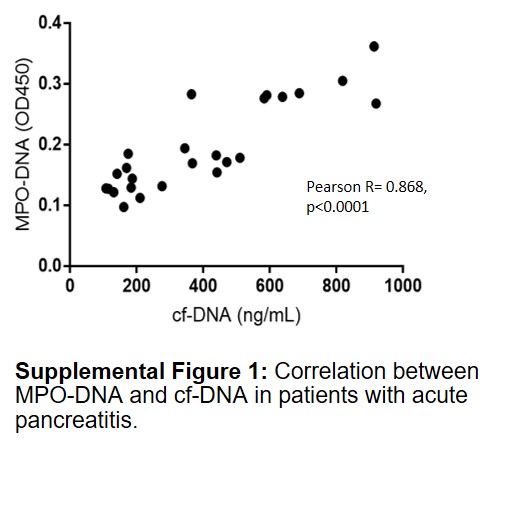

Supplement: Supplementary file 2 [file Image_1.JPEG]

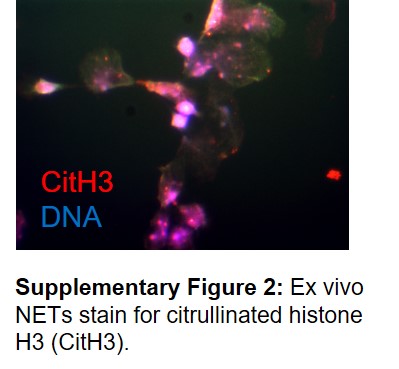

Supplement: Supplementary file 3 [file Image_2.JPEG]

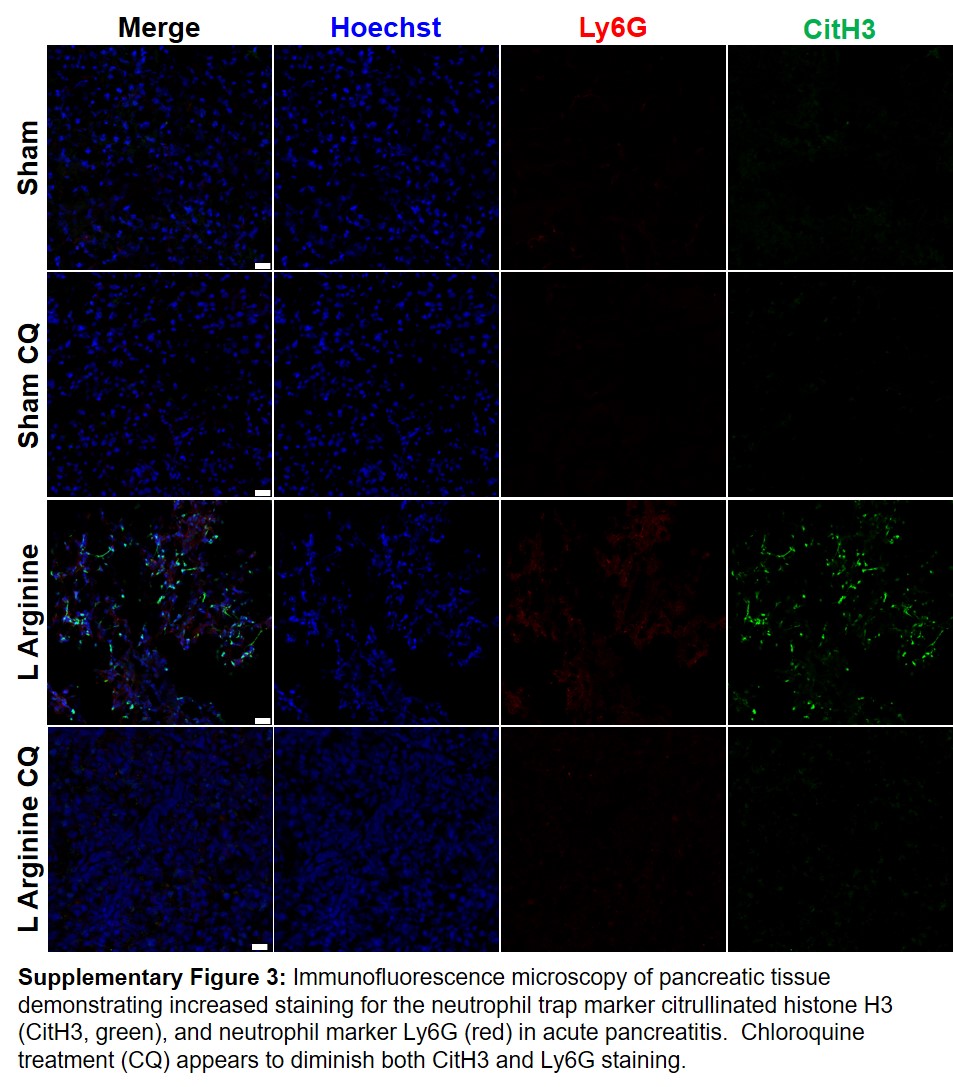

Supplement: Supplementary file 4 [file Image_3.JPEG]

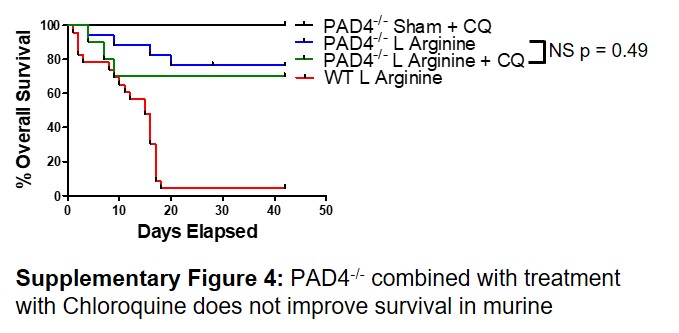

Supplement: Supplementary file 5 [file Image_4.JPEG]
